# The SABRTooth feasibility trial protocol: a study to determine the feasibility and acceptability of conducting a phase III randomised controlled trial comparing stereotactic ablative radiotherapy (SABR) with surgery in patients with peripheral stage I non-small cell lung cancer (NSCLC) considered to be at higher risk of complications from surgical resection

**DOI:** 10.1186/s40814-016-0046-2

**Published:** 2016-02-01

**Authors:** M. P. Snee, L. McParland, F. Collinson, C. M. Lowe, A. Striha, D. R. Baldwin, B. Naidu, D. Sebag-Montefiore, W. M. Gregory, J. Bestall, J. Hewison, S. Hinsley, K. Franks

**Affiliations:** 1 Department of Clinical Oncology, Leeds Cancer Centre, Leeds Teaching Hospitals NHS Trust, Beckett Street, Leeds, LS9 7TF UK; 2 Clinical Trials Research Unit (CTRU), Leeds Institute of Clinical Trials Research, University of Leeds, 71-75 Clarendon Road, Leeds, LS2 9PH UK; 3 Respiratory Medicine Unit, David Evans Research Centre, Nottingham University Hospitals and University of Nottingham, Hucknall Rd, Nottingham, NG5 1PB UK; 4 School of Clinical and Experimental Medicine, University of Birmingham, Birmingham, Edgbaston B15 2TT UK; 5 Leeds Institute of Health Sciences, Faculty of Medicine and Health, University of Leeds, 101 Clarendon Rd, Leeds, LS2 9LJ UK; 6 Leeds Institute of Cancer and Pathology, Faculty of Medicine and Health, University of Leeds, Beckett Street, Leeds, LS9 7TF UK

**Keywords:** NSCLC, Lung cancer, SABR, Surgery, Radiotherapy, Feasibility, Randomised, Stereotactic

## Abstract

**Background:**

Stage I non-small cell lung cancer (NSCLC) is potentially curable, and surgery is considered to be the standard of care for patients with good performance status and minimal co-morbidity. However, a significant proportion of patients with stage I NSCLC have a poorer performance status and significant medical co-morbidity that make them at higher risk of morbidity and mortality from surgery.

Stereotactic ablative radiotherapy (SABR), which uses modern radiotherapeutic techniques to deliver large doses of radiation, has shown superiority over conventional radiotherapy in terms of local control and toxicity and is a standard of care for patients with stage I NSCLC who are at too high risk for surgery. However, it is not known whether surgery or SABR is the most effective in patients with stage I NSCLC who are suitable for surgery but are less fit and at higher risk surgical complications. Previous randomised studies have failed to recruit in this setting, and therefore, a feasibility study is required to see whether a full randomised control trial would be possible.

**Methods/design:**

SABRTooth is a UK-based, multi-centre, open-label, two-group individually (1:1) randomised controlled feasibility study in patients with peripheral stage I NSCLC considered to be at higher risk from surgical resection. The study will assess the feasibility of conducting a definitive large-scale phase III trial. The primary objective is to assess recruitment rates to provide evidence that, when scaled up, recruitment to a large phase III trial would be possible; the target recruitment being 54 patients in total, over a 21-month period. There are multiple secondary and exploratory objectives designed to explore the optimum recruitment and data collection strategies to help optimise the design of a future phase III trial.

**Discussion:**

To know whether SABR is a better, equivalent or inferior alternative to surgery for higher risk patients is a key question in lung cancer. Other studies comparing SABR to surgery have closed early due to poor recruitment, and therefore, the SABRTooth feasibility study has been designed around the UK National Health Service (NHS) cancer pathway incorporating many design features in order to maximise recruitment for a future definitive phase III trial.

**Trial registration:**

controlled-trials.com ISRCTN13029788

## Background

### Non-small cell lung cancer (NSCLC)

Stage I NSCLC is potentially curable, and surgery is considered the standard of care for patients with good performance status and minimal co-morbidity, with 5-year overall survival (OS) of around 60 % [1]. However, a significant proportion of patients with stage I NSCLC are not suitable for surgery because of poor fitness, often related to the presence of significant medical co-morbidities including chronic obstructive pulmonary disease (COPD) and cardiovascular disease. For instance, the National Lung Cancer Audit (NLCA) database of lung cancer treatment in England and Wales from 2008 to 2012 showed that the resection rate for patients with histologically confirmed NSCLC was 20.8 and 21.4 % in those patients aged <65 and 65–74, respectively; for patients aged >75, the rate was 13.9 % [2]. An analysis of the same database shows that 30- and 90-day mortality following surgery rise steeply with age [3]; 90-day mortality being 6.4 % for 70-year-olds versus 43 % for 80-year-olds, a deteriorating performance status is also a factor influencing the outcome [4].

An alternative, potentially curative, treatment to surgery is conventionally fractionated radical radiotherapy [5]. However, the results of conventional fractionated radical radiotherapy (using 20–33 fractions of radiation at a daily dose of 2–2.75 Gy to a total dose of 55–66 Gy) are poor. A meta-analysis reported OS at 2- and 5-year rates of 52 and 19 % [6], respectively, with a mean local recurrence rate of 40 % [7]. Though the CHART trial of hyper-fractionated radiotherapy and modelling studies have shown a steep dose–response curve for local control of NSCLC [4, 8], a trial comparing 60 with 74 Gy, albeit combined with concurrent chemotherapy, was stopped due to increased mortality in the high-dose arm. Thus, 60–66 Gy in 2 Gy per fraction has been the standard of care for NSCLC in many countries; in the UK, a hypofractionated regime of 55 Gy in 20 fractions is also used. Concerns regarding toxicity often preclude patients with significant co-morbidity from being offered conventionally fractionated radical radiotherapy. However, stereotactic ablative radiotherapy (SABR) or stereotactic body radiotherapy (SBRT), which uses modern radiotherapeutic techniques to deliver large doses of radiation in 3–8 fractions to a total in excess of 100 Gy biologically equivalent dose (BED), has shown superiority (albeit in uncontrolled series) to conventional radiotherapy in terms of local control and toxicity [6, 7].

SABR is highly accurate in its delivery and conformal with the irradiated volume (planning treatment volume—PTV); consisting of an individually determined margin for tumour movement, a very small margin added for daily set-up error as image guidance is used during the treatment and with no margin applied for microscopic spread. Thus, the proportion of normal lung irradiated to significant dose is much lower for SABR compared with conventionally fractionated radical radiotherapy.

Hence, SABR is a low-risk treatment in the majority of patients, even those with severe pulmonary morbidity (excluding those with interstitial pulmonary fibrosis). A meta-analysis has shown a 5-year survival of 42 % [6] and local recurrence of less than 10 % [7]. A study of the treatment of early stage NSCLC in a large region of the Netherlands showed that as SABR was introduced, the proportion of patients offered no specific anti-cancer treatment fell; those offered radical radiotherapy rose without affecting the number of patients receiving surgery, accompanied by an improvement in OS for early stage NSCLC [9]. These data strongly imply that SABR is a good option for the majority of patients with medically inoperable NSCLC.

### Patient population

A meta-analysis compared SABR with surgery in cohorts of patients, with adjustment for age and medical operability, and found similar outcomes for mortality and tumour control [10]. However, no adequately powered randomised trial comparing the two modalities has been successful.

Worldwide, there have been three trials comparing surgery and SABR: ROSEL (NCT00687986), STARS (NCT00840749) and ACOSOG–RTOG (NCT01336894), all of which closed early due to failure to recruit. In ROSEL, this was thought to be due to lack of support from the surgical community as it included medically operable patients [11]. The STARS trial was also limited to patients fit for conventional surgery (lobectomy or pneumonectomy), whilst the RTOG trial was limited to patients at high risk of lobectomy, where the planned surgery was sub-lobar (wedge or segmental) resection. An intention-to-treat pooled analysis of the ROSEL and STARS trial was recently published. In the STARS trial, of the 234 patients screened, only 36 were enrolled and randomly assigned and only 22 patients were enrolled into the ROSEL study [12]. The results of this analysis have shown an overall advantage, in terms of 3-year survival, for SABR; however, because of the limited power of the study, this result cannot be considered definitive.

Previously, the only randomised trial of surgery versus radiotherapy for NSCLC recruited 58 patients from 1954 to 1958 and was published in 1963; this showed a 4-year survival for surgery of 23 versus 7 % for radiotherapy, although the difference was not statistically significant [13]. Therefore, bearing in mind the difficulty of carrying out a randomised trial of surgery versus radiotherapy, a multidisciplinary group was established to produce the protocol for the SABRTooth trial. This multidisciplinary group designed the protocol around the well-established referral pathway for cancer patients in the UK where all cancer patients’ cases are discussed in a multidisciplinary team (MDT) meeting before a treatment decision is made. At least one representative of all clinical specialities (pathologists, surgeons, chest physicians, oncologists, cancer nurses and radiologists) involved in lung cancer diagnosis and management is present to provide a balanced treatment decision.

The aim was to produce a pragmatic protocol based on everyday practice such that SABR could be delivered using any approved technique, and the choice of surgical procedure (lobectomy, anatomical segmentectomy or wedge resection) was left to the discretion of the treating thoracic surgeon.

Subsequently, a small number of established lung cancer centres and their referral units were chosen for this feasibility trial with a trial workshop and launch meeting to help get “buy-in” from surgeons, respiratory physicians and oncologists from each centre.

## Methods/design

### Trial aims and objectives

The SABRTooth trial aims to determine the feasibility and acceptability of conducting an adequately powered definitive phase III randomised controlled trial (RCT) comparing surgery with SABR in patients with peripheral stage I NSCLC deemed at higher risk of complications from surgical resection.

### Trial design

**Fig. 1:**
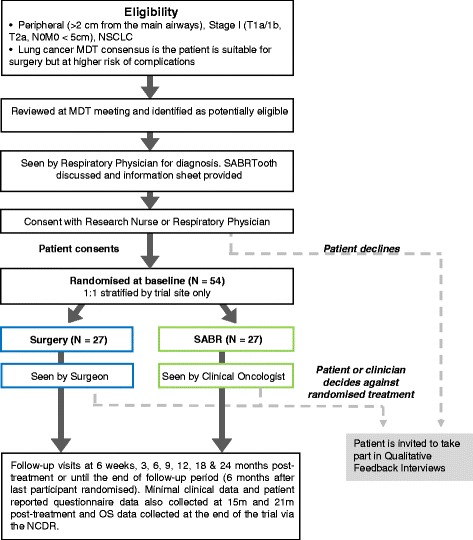
SABRTooth trial schema

SABRTooth is a UK-based, multi-centre, open-label, two-group individually randomised controlled feasibility study targeted at patients with peripheral stage I NSCLC considered at higher risk from surgical resection. Patients will be randomised (1:1) to either SABR or surgical resection (Fig. 1). Due to the different treatment modalities, it is not possible to blind patients or clinicians to treatment allocation.

### Patient and public involvement (PPI)

Given the known difficulties in recruiting patients to RCTs where the two treatment options are very difficult, PPI has been critical in the development of the trial. We have had patient and patient’s relatives input into the protocol design, lay summary patient information leaflets and held an initial trial workshop and trial launch meeting. In addition, we have two PPI members on the trial management group (TMG) and a further PPI member on the trial steering committee (TSC).

### Trial population

Those patients considered at higher risk of complications from surgical resection are defined as “a level of fitness that could lead to a greater than average morbidity or mortality from surgery”. This includes those patients with poor lung function who are at risk of increased post-operative dyspnoea that would be sufficient to be unacceptable to them (this is subjective and will vary from patient to patient) and patients who have an increased risk of cardiovascular morbidity that might lead to a perioperative complication or death.

In order to maximise recruitment, the protocol does not specify what constitutes high risk but leaves it to the judgment of the individual MDT. However, we have produced guidelines (Fig. 1: SABRTooth trial schema; Table 1) to ensure appropriate patients are considered for the trial. We also intend to collect data on the number of patients considered for the trial but not invited to enter because their risk is deemed too high or within acceptable limits for surgery, along with the reason(s) for why they were not considered suitable.

**Table 1:** Guidance for definition of higher risk from surgical complications

Group A		
Suitable for surgery—but at higher risk of complications compared to group B (potentially eligible for SABRTooth)	▪ CPEX—VO_2_ max 10–15 L/kg/min ▪ ISWT—walk 250–400 m ▪ Mortality risk from Nottingham score: 6–20 % at 90 days	The patient can be approached for the trial if they meet one or more of these criteria
Group B		
Suitable for surgery—lower risk of complications	▪ CPEX—VO_2_ max >15 L/kg/min, anaerobic threshold ▪ ISWT—walk >400 m and without significant desaturation ▪ Predicted post-operative FEV_1_ > 50 % ▪ Mortality risk from Nottingham score <6 % at 90 days for lobectomy (it is not anticipated that patients will need a pneumonectomy in this group of peripheral cancers).	Not suitable for the trial
Group C		
Unsuitable for surgery as predicted risk of complications is too high	▪ CPEX—VO_2_ max <10 L/kg/min ▪ ISWT—walk <250 m and significant desaturation ▪ Pre-operative FEV_1_ < 30 % ▪ Mortality risk from Nottingham score >20 % at 90 days for lobectomy (it is not anticipated that patients will need a pneumonectomy in this group of peripheral cancers). ▪ Reduced ejection fraction (e.g. <40 %) or evidence of ongoing myocardial ischemia ▪ Recent cerebrovascular event (e.g. within 3 months of planned surgery)	Not suitable for the trial

We have suggested the above criteria for all groups to assist patient selection. However, as there are other individual contributing factors, the final decision on whether the patient is suitable for the trial will rest with the local MDT

### Overall primary objectives

- To quantitatively assess the recruitment rate in order to provide evidence that when recruitment rates are scaled up, recruitment to a large phase III trial would be possible

There will be a 21-month recruitment period. In order to demonstrate that recruitment, targets for the planned phase III trial can be met within an adequate timeframe; a “steady state” of recruitment must be observed. Formal monitoring of recruitment will begin 6 months after the start of recruitment (allowing a run-in period for set-up), where an average of three patients per month must be randomised over the remaining recruitment period in order to demonstrate a “steady state” of recruitment.

### Secondary objectives

- To quantitatively assess uptake of allocated treatment arm (surgery/SABR) as a measure of acceptability of the trial and appropriateness of patients recruited
- To determine the number of patients screened and identified as eligible per month, to provide evidence of appropriate numbers of patients, to meet the sample size requirements of a future phase III trial
- To explore reasons for non-participation of eligible patients and participants not undergoing their allocated treatment procedure
- To assess the feasibility of collecting the quality of life (QoL) data and determine the optimal frequency of data collection for the planned phase III trial, specifically for the Euro-Quality of Life 5D utility score (EQ-5D) and Euro-Quality of Life visual analogue scale (EQ-VAS), Cancer Quality of Life Questionnaire (QLQ-C30), Lung Cancer-Specific Quality of Life Questionnaire (QLQ-LC13) and a medical resource use and societal–economic questionnaire
- To obtain EQ-5D utility estimates to inform the sample size calculations for the phase III trial

### Exploratory objectives

- To qualitatively explore in a cohort of patients acceptability of the trial (i.e. reason for trial non-participation and participant refusal of allocated treatment) to assist in optimisation of recruitment strategies employed for a future definitive phase III trial
- To explore participant recruitment pathways at both treatment centres and referral units in order to assist in recruitment strategy planning for a future study
- To explore the use of newly available tools (Nottingham risk score) in defining patients at a higher risk from surgical resection across the six trial sites in order to assist in optimising recruitment for a future study
- To monitor the 30-/90-/180-day mortality rates overall and in both treatment arms (surgery/SABR) and overall survival (OS) at the end of the study

### Sample size

As the study is designed to assess the feasibility of conducting a definitive large-scale phase III trial, a formal power calculation is not considered appropriate as effectiveness is not being evaluated.

For this feasibility study, we plan to recruit 54 patients in total (i.e. 27 to each treatment arm) from six UK hospitals, four tertiary treatment centres and two smaller referral units, over a 21-month recruitment period.

Achieving the recruitment targets within the feasibility study will help demonstrate that, when recruitment rates are scaled up, recruitment into a large-scale phase III trial is feasible. It has been estimated that 690 patients are required for the phase III trial to have 80 % power to show non-inferiority (NI) of SABR to surgery, where this is defined as SABR being less than 8 % worse in terms of 3-year OS (derived from an in-house simulation programme which accounts for the likely excess early death rate in the surgery arm in comparison with the SABR arm). This sample size also yields >97 % power for a similar 10 % NI margin for difference in mean quality-adjusted life years (QALYs) per arm and greater than 80 % power to show superiority.

In order to demonstrate the feasibility of recruitment within this feasibility study, we are aiming to recruit on average three patients per month from four UK large treatment centres and two smaller referral units. This equates to 36 patients per year (i.e. 9 patients from each of the 4 large treatment centres). Including these 4 established centres (and their referral units) within a subsequent phase III trial, recruiting at the same rate, 180 patients could be recruited over a 5-year period. We would also aim to include an additional 15 UK large treatment centres, plus their associated referral units. If these 15 centres recruited at 75 % of the rate of the centres within the feasibility study, an additional 100 patients could be recruited a year; 500 patients over a 5-year period. Thus, with approximately 19 large treatment centres and their associated referral units, a target of 690 patients over a 5-year period would be feasible, which equates to a rate of 138 patients per year.

Additionally, a number of international groups including Australia (TROG) and Europe have confirmed interest in the study and are keen to be involved. International collaboration for the phase III trial would increase the trial population considerably and reduce the recruitment period significantly.

### Recruitment

SABRTooth opened to recruitment in July 2015.

The recruitment period is 21 months, and all participants will be followed up until 6 months after the last patient has been randomised. Participants will be recruited from six UK hospitals in total, a mixture of tertiary treatment centres and smaller referral units. All treatment centres are established lung cancer surgical centres with established SABR programmes for lung cancer and a proven track record of recruitment into clinical trials.

We recognise the challenge of avoiding clinician bias and the need to describe equipoise between the two very different treatment interventions. To address this, the respiratory physician and research nurse will introduce the trial to avoid any bias. We have assessed existing evidence in optimising recruitment to trials and held mock patient interview scenarios and used these as a basis to develop a training guide for recruiters on how best to approach and discuss the trial with patients [14, 15]. A careful explanation of the risks and benefits of the two treatment interventions is crucial; such risks will be clearly explained to interested patients in an unbiased and fair way, assisted by written trial-specific patient information. Potential patients for this study will be identified by expert lung cancer teams through the appropriate MDT meeting.

### Randomisation

Following confirmation of written informed consent and eligibility, patients will be randomised using a 24-hour telephone or web-based system based at the Leeds Institute of Clinical Trials Research (Leeds, UK). Patients will be randomised using stratified permuted blocks to either surgery or SABR (1:1), stratified for recruiting site only. Patient randomisation is to take place as close to the intervention start date as possible and, in line with National Health Service (NHS) guidelines, is aimed to be done no more than 31 days prior to treatment start date.

### Pre-treatment investigations

Patients must have had a pathological and/or clinical and radiological diagnosis of stage I NSCLC. Pathological confirmation prior to randomisation is encouraged, but patients without histology, providing the putative tumour has demonstrated growth on serial radiological imaging (preferably, on CT, but if the lesion is clearly evaluable on chest X-ray, this will be acceptable) and is positron emission tomography (PET) avid (showing FDG avidity greater than the mediastinal blood pool), will be eligible (Table 2).

**Table 2:** SABRTooth trial inclusion and exclusion criteria

Inclusion criteria
1. Histological and/or clinical and radiological diagnosis of NSCLC 2. Primary tumour characteristics i. Peripherally located tumour as defined in the RTOG 0236 and UK SABR consortium guidelines. This states that the tumour must be more than 2 cm in axial diameter from a major airway = “No Fly Zone”. This includes the trachea, carina, right and left main bronchus and extends to the bifurcation of the right upper, right middle, right lower, left upper and left lower lobe bronchioles. Maximal axial diameter ≤5 cm measured on lung windows on computed tomography (CT). 3. No evidence of hilar or mediastinal lymph node involvement. Any hilar or mediastinal lymph nodes that are either PET positive or >1 cm in short diameter must be sampled by endo-bronchial ultrasound or oesophageal endoscopic ultrasound or mediastinoscopy and demonstrate negative cytology and/or pathology. 4. The local lung cancer MDT is of the opinion that a patient is considered suitable for either surgical resection or SABR treatment AND also to be at higher risk complications from surgical resection. 5. Age ≥18 6. Female patients must satisfy the investigator that they are not of childbearing potential or not pregnant (i.e. be willing to undergo a pregnancy test within 72 hours of surgery or day 1 of SABR) or are not of childbearing potential. 7. Able and willing to provide written informed consent
Exclusion criteria
1. Previous radiotherapy within the planned treatment volume 2. History of clinically significant diffuse interstitial lung disease 3. Any history of concurrent or previous invasive malignancy that in the opinion of the investigator could impact on trial outcomes 4. Clinical or radiological evidence of metastatic spread 5. History of psychiatric or addictive disorder, or other medical condition that, in the opinion of the investigator, would preclude the patient from meeting the trial requirement. 6. Previous systemic therapies, including targeted and experimental treatments, for their current lung cancer diagnosis

The following tests are required to determine eligibility:

- History and examination
- Full body PET-CT (within 8 weeks prior to randomisation)
- Pregnancy test (if woman of childbearing potential)
- Assessment of Eastern Cooperative Oncology Group performance status (ECOG PS), weight, MRC dyspnoea score, Charlson co-morbidity index [16]
- Pulmonary function tests (FEV_1_, FVC, KCO and DLCO/VA)
- Calculated predicted post-resection lung function
- FBC and serum biochemistry (FBC U+E, LFT, calcium)
- ECG
- Cardio-pulmonary exercise testing (CPEX) and/or Incremental Shuttle Walk Test (ISWT)
- Thoracoscore, calculated global risk score [17]
- Nottingham risk score [3]

The following investigations and assessments may be carried out prior to randomisation, if clinically indicated:

- Cardiac tripartite risk assessment
- Cardiology review if the patient has an active cardiac condition, ≥3 risk factors or poor cardiac functional capacity
- Split lung function testing can be used if a ventilation or perfusion mismatch is suspected

### Follow-up

The planned duration of the trial follow-up is until 6 months after the last participant is randomised. Follow-up frequency will be in line with current NHS practice with routine follow-up visits and data collection at 6 weeks, 3, 6, 9, 12, 18 and 24 months post-treatment. Minimal clinical data will also be recorded at 15 and 21 months post-treatment; patient-reported questionnaires due to be completed at these time points will be administered via post. OS data will be captured again at the end of the study for all participants via the National Cancer Data Repository (NCDR) (Fig. 1).

Data collected at follow-up will include the following:

- Post-operative/post-SABR complications and severity
- Further anti-cancer therapy
- Patient status (alive or dead)
- ECOG PS, weight, MRC dyspnoea scale score
- Details of any local or distant recurrence, including date and site of recurrence and method of diagnosis
- Details of any new primary cancer diagnoses including date and site of recurrence and method of diagnosis

Follow-up imaging by CT scan will be performed as per local practice, and it is anticipated that this will be at 6, 12 and 24 months post-treatment. Other imaging is not mandated and should be performed as clinically indicated.

### Trial organisation and administration

The SABRTooth trial is funded by the National Institute for Health Research (NIHR), Research for Patient Benefit (RfPB) Programme (PB-PG-0613-31114), and is sponsored by Leeds Teaching Hospitals NHS Trust.

Trial supervision will be established according to the principles of Good Clinical Practice (GCP) and in line with the relevant Research Governance Framework within the UK and through adherence with CTRU standard operating procedures (SOP). A core internal project team, TMG and TSC will be established, with the TSC performing a dual role and acting as the data monitoring and ethics committee (DMEC). Overall data and trial management will be provided by the Clinical Trials Research Unit (CTRU) (University of Leeds, UK).

### Qualitative interviews

The trial includes embedded qualitative exploration of the reasons for non-participation in the SABRTooth study. As the two treatments are very different, patients may have a strong preference for either surgery or SABR or may feel uncomfortable to have a decision between such different treatment options being taken out of their hands. What patients understand, perceive and feel about how the SABRTooth study and how it was presented to them and their expectations of study burden will be explored in qualitative feedback interviews. Participants who agree to participate in the study but subsequently do not take up their treatment allocation after being randomised will also be invited to take part in a feedback interview (Fig. 1). Understanding why patients choose not to participate or do not take up their treatment allocation will be crucial in assisting optimal recruitment methods and ensuring the most appropriate design of any subsequent phase III study.

#### Quality of life

Participants will be asked to complete questionnaires at baseline, day 1 of treatment, 6 weeks post-treatment and then 3 monthly until 24 months post-treatment or until the end of the trial follow-up period (6 months after the last patient has been randomised). Questionnaires to be completed include the following:

- EQ-5D™ and EQ-VAS™
- EORTC QLQ-C30 and QLQ-LC13 (not collected at 15- and 21-month time points)
- Baseline and follow-up resource use and societal–economic questionnaires

Questionnaires will be completed by participants at their standard clinic visits, i.e. baseline; day 1 of treatment; 6 weeks post-treatment; and 3, 6, 9, 12, 18 and 24 months post-treatment. Questionnaires due to be completed at 15 and 21 months post-treatment will be administered via post.

The frequent collection of quality of life (QoL) data within this feasibility study is necessary in order to assess the burden to patients. This will be assessed by monitoring compliance rates in terms of proportion of questionnaires returned and completed and will inform the optimal frequency of data collection for the planned subsequent phase III trial. Averaged QALYs are intended as a co-primary endpoint for the planned phase III trial, as such, determining the optimal frequency of EQ-5D data collection within this feasibility study is crucial. Utility estimates will be derived from the EQ-5D data collected within this feasibility study and used to inform the power calculations for the QALY co-primary endpoint.

### Health economics

As this is a feasibility study, a formal cost analysis or cost-effectiveness analysis will not be performed. The resource use and societal–economic data will be analysed and reported using descriptive statistics of frequency and quantity of specific resources used on average by patients. The objective will be to identify the main drivers of difference in resource use and out-of-pocket expenses between the two arms. Compliance and missing data will also be described. These data will inform an efficient design of the patient questionnaires in the subsequent phase III study.

### Statistical methods and analysis

Statistical analysis will be the responsibility of the SABRTooth CTRU trial statistician. A full statistical analysis plan will be written before any formal analyses are undertaken. As this is a feasibility study looking at recruitment potential, the analysis will be performed through descriptive statistics rather than formal hypothesis testing.

The analysis of the primary endpoint and all secondary endpoints relating to recruitment and withdrawals from the trial will take place at the end of the 21-month recruitment period. Final analysis of all other endpoint data will be carried out 6 months after the final participant has been randomised.

The primary endpoint analysis will be based on the population of participants recruited within the formal monitoring period. All further analysis will be carried out on the intention-to-treat (ITT) population defined as all participants randomised to the trial, regardless of adherence to the protocol, withdrawal of consent or losses to follow-up. Participants will be included within the treatment arm to which they were randomised.

An interim analysis will take place approximately midway through the study and be reported to the TSC. The aim of the interim analysis is to evaluate and monitor the key study objectives (i.e. recruitment rates, number of participants taking up their treatment allocation, feedback from the qualitative interviews), as well as expected serious complications (SCs) and unexpected serious complications (USCs) and the delivery time of surgery/SABR post-randomisation. The TSC will have the opportunity to raise any concerns with the trial progression/running allowing the trial team to improve processes going forward.

#### Primary endpoint analysis

- Recruitment rates over the whole 21-month recruitment period will be reported overall and by recruiting site. The average recruitment rate per month and in total over the formal monitoring period will be reported in order to evaluate the primary objective of the study.

#### Secondary endpoint analysis

- The total number of patients considered for the trial and assessed as being eligible will be reported by recruiting site along with reasons for non-randomisation.
- The proportion of participants undergoing their allocated treatment procedure (surgery/SABR) will be summarised overall, by treatment arm and by recruiting site, along with reasons for non-compliance.
- The proportion of QoL questionnaires returned and completed at each data collection timepoint will be reported overall, by treatment arm and by recruiting site. EQ-5D utility scores and standard deviations will be derived for each participant in line with the EQ-5D user guide. Average utility scores over follow-up will be presented for each treatment arm and overall and used to inform the sample size calculations for the planned phase III trial.

#### Exploratory data analysis

- The feedback interviews will be professionally transcribed verbatim and managed with help of NVivo. The data will be analysed using inductive thematic analysis [18, 19]. The analysis will be further refined by using a constant comparison and contrastive approach and looking for negative cases in order to examine for similarities and differences within and between the patients in different sites and within and between patient groups.
- Descriptive summaries of the participant recruitment pathways at the individual sites and site criteria for identifying patients at a higher risk from surgical resection will be reported. Intended pathways and criteria used at the start of recruitment and subsequent changes during recruitment will be described.
- The 30-, 90- and 180-day mortality rates overall and in each treatment arm (measured post-treatment) will be summarised, and 95 % confidence intervals will be constructed. Given that these rates will be based on a limited sample, they will be treated as exploratory and used only to compare with recent figures in the literature rather than influence the sample size for the planned phase III trial.
- OS rates as obtained via the NCDR will be reported but an analysis formally comparing the two treatment arms will not be performed due to the lack of power within this feasibility study. By collecting OS data on all participants within this study, this may then be used in conjunction with the OS data generated from the planned phase III trial in a formal meta-analysis which will add to the overall evidence base for these two interventions.
- Rates of related complications, serious complications and unexpected serious compliances will be reported by treatment arm and overall, along with the average and range of delivery times of surgery and SABR post-randomisation.

## Discussion

To know whether SABR is a better, equivalent or inferior alternative to surgery for higher risk patients is a key question in lung cancer. The primary aim of this feasibility study is to demonstrate that, over 21 months, enough patients can be randomised from 6 centres to indicate that a phase III trial recruiting 690 patients over 5 years in 20 UK centres would be feasible. The multiple secondary and exploratory endpoints will help determine the most optimum recruitment and data collection strategies and help optimise the design of a future phase III trial where the key aims would be to show NI of SABR to surgery and determine the relative costs of the two treatments and their effects on quality of life. Other studies comparing SABR to surgery have closed early due to poor recruitment, and therefore, the SABRTooth trial is a pragmatic study based on everyday practice in hospitals managing lung cancer. Considerable effort has been made to try and maximise recruitment.

- The SABRTooth feasibility study has been designed around the UK NHS cancer pathway for managing lung cancer.
- Neither histological proof nor exact quantitative measurement of risk for surgery is mandated for entry into the trial.
- The protocol has been developed through national lung cancer meetings over the past 3–4 years to promote awareness to the UK clinical oncologists, respiratory physicians and thoracic surgeons.
- A strong MDT with representatives of all concerned medical specialities involved in the management of lung cancer has developed the protocol.
- A small number of centres with highly motivated clinicians have been selected.
- PPI has been critical to the development of the protocol, lay summary and patient information leaflets.
- Mock patient interview scenarios with constructive feedback have been produced to provide a guide on how to approach and discuss the trial with patients.
- The trial is introduced to patients by a “neutral” respiratory physician and then, if the patient is interested in the study, consent is obtained by a lung cancer trials nurse.
- Approached patients who decline randomisation or their allocated treatment arm are offered an interview to explore their reasons for not entering the trial. Lessons learnt can then be used to try and improve recruitment.

Therefore, we believe that such a well-designed feasibility study in this clinical scenario will address very important issues of clinician and patient equipoise. We hope that, if this study is successful, we will be able to progress to a national randomised phase III study.

## Abbreviations

BED: biologically equivalent dose
CT: computerised tomography
CTRU: Clinical Trials Research Unit
CPEX: cardio-pulmonary exercise testing
ECG: electrocardiogram
ECOG PS: Eastern Cooperative Oncology Group performance status
EORTC: European Organization for Research and Treatment of Cancer
EQ-5D™: Euro-Quality of Life 5D utility score
EQ-VAS™: Euro-Quality of Life visual analogue scale
GCP: Good Clinical Practice
ISWT: Incremental Shuttle Walk Test
ITT: intention-to-treat
MDT: multidisciplinary team
NCDR: National Cancer Data Repository
NLCA: National Lung Cancer Audit
NHS: National Health Service
NI: non-inferiority
NIHR: National Institute for Health Research
NSCLC: non-small cell lung cancer
PET: positron emission tomography
QALY: quality-adjusted life year
QLQ-C30: Cancer Quality of Life Questionnaire
QLQ-LC30: Lung Cancer-Specific Quality of Life Questionnaire
QoL: quality of life
OS: overall survival
RCT: randomised controlled trial
SABR: stereotactic ablative radiotherapy
SC: serious complication
SOP: standard operating procedure
TMG: trial management group
TSC: trial steering committee
USC: unexpected serious complication
