# Erratum to: The SABRTooth feasibility trial protocol: a study to determine the feasibility and acceptability of conducting a phase III randomised controlled trial comparing stereotactic ablative radiotherapy (SABR) with surgery in patients with peripheral stage I non-small cell lung cancer (NSCLC) considered to be at higher risk of complications from surgical resection

**DOI:** 10.1186/s40814-016-0095-6

**Published:** 2016-09-12

**Authors:** M. P. Snee, L. McParland, F. Collinson, C. M. Lowe, A. Striha, D. R. Baldwin, B. Naidu, D. Sebag-Montefiore, W. M. Gregory, J. Bestall, J. Hewison, S. Hinsley, K. N. Franks

**Affiliations:** 1 Department of Clinical Oncology, Leeds Cancer Centre, Leeds Teaching Hospitals NHS Trust, Beckett Street, Leeds, LS9 7TF UK; 2 Clinical Trials Research Unit (CTRU), Leeds Institute of Clinical Trials Research, University of Leeds, 71-75 Clarendon Road, Leeds, LS2 9PH UK; 3 Respiratory Medicine Unit, David Evans Research Centre, Nottingham University Hospitals and University of Nottingham, Hucknall Rd, Nottingham, NG5 1PB UK; 4 School of Clinical and Experimental Medicine, University of Birmingham, Birmingham, Edgbaston B15 2TT, UK; 5 Leeds Institute of Health Sciences, Faculty of Medicine and Health, University of Leeds, 101 Clarendon Rd, Leeds, LS2 9LJ UK; 6 Leeds Institute of Cancer and Pathology, Faculty of Medicine and Health, University of Leeds, Beckett Street, Leeds, LS9 7TF UK

## Erratum

After publication of the original article [1], it came to the authors’ attention that a source of funding had been inadvertently omitted from the Acknowledgements section; support from Yorkshire Cancer Research should have mentioned originally.

## References

[1] Snee MP, McParland L, Collinson F, Lowe CM, Striha A, Baldwin DR (2016). The SABRTooth feasibility trial protocol: a study to determine the feasibility and acceptability of conducting a phase III randomised controlled trial comparing stereotactic ablative radiotherapy (SABR) with surgery in patients with peripheral stage I non-small cell lung cancer (NSCLC) considered to be at higher risk of complications from surgical resection. Pilot Feasability Studies.

